# Supplementary material for: Research Implications for Future Telemedicine Studies and Innovations in Diabetes and Hypertension—A Mixed Methods Study
Source: Nutrients. 2020 May 8;12(5):1340. doi: 10.3390/nu12051340 (PMC7284383; doi:10.3390/nu12051340)
Supplement: Supplementary file 1 [file nutrients-12-01340-s001.pdf]

Table S1. Identified categories and sub-categories on future research implications.

#	Category	Sub-category
I	Need for high quality studies including specific outcome measures	Study designs<br>Costs /cost-effectiveness<br>Long-term effects<br>User satisfaction and technology acceptance<br>Adherence to reporting standards
II	Need for comprehensive technology assessment Data	Understanding the prerequisites, mechanisms and combinations<br>Comprehensive assessment of features and functionalities
III	Need for in-depth considerations of patients' characteristics and more diverse study populations	
IV	Ethics & Safety	
V	Translation and implementation strategies	

Categories and sub-categories inferred in the qualitative content analysis are depicted.

Table S2. Occupational background of study sample.

Occupational background	n total	n considered in subsequent analysis
**Working in health care**	**172**	**77**
Ergotherapy	77	24
Physical therapy	52	23
Medical care as a doctor (unspecified)	8	8
Nutritional therapy	7	3
Speech therapy	5	3
Therapy (unspecified)	5	3
Management of a therapeutic center	5	2
Dietitian	3	2
Dental medicine	3	3
Psychological therapy	2	2
Adiposity coaching	1	1
Health coaching (unspecified)	1	0
Psychosocial coaching	1	0
Eye therapy	1	1
Pediatrics	1	2
**Working in medical device regulation**	**13**	**7**
**Working in research**	**7**	**4**
Teaching position	4	1
Research	1	1
**Unspecified**	**2**	**2**
**Working in financing of health care**	**1**	**1**
**Else (Working at an NGO in healthcare)**	**1**	**0**

Left column shows the occupational background (bold) with potential subcategory (normal). The second column shows the number of respondents (n) considered within the subsequent analyses. An overall n >188 is due to the fact that multiple imputations where possible for the occupational background.

Table S3. Previous experience with telemedicine among the participants.

	n total	n considered for analysis
**Telemedicine development (overall n = 127)**		
No experience at all	94	52
Sporadic experience	21	10
Continuous experience	12	12
**Use of telemedicine in patient care (overall n = 130)**		
No experience at all	58	36
Sporadic experience	54	32
Continuous experience	18	12
**Counselling patients on telemedicine use (overall n = 128)**		
No experience at all	67	36
Sporadic experience	45	28
Continuous experience	16	14
**Telemedicine evaluation (overall n = 128)**		
No experience at all	81	42
Sporadic experience	37	26
Continuous experience	10	8

Differences in n total and n considered for analysis are due to dropouts after page 2. Overall n represents the sum of the total n and differs from one category to another due to no response. A dichotomous variable for previous vs. no previous experience with telemedicine was used as the independent variable for the t-test. In a second step, dichotomous variables for four types of previous experience with telemedicine were computed: experience in development and evaluation of telemedicine, as well as experience using telemedicine in patient care and advising patients how to use telemedicine.

Table S4. Ranking of research needs.

Research need	n	mean	variance
1) Implementation strategies	46	5,04	0,92
2) Adequate tailoring of applications to patient needs	45	4,64	1,87
3) Consideration of data security	45	5,25	1,27
4) Consideration of patient satisfaction	45	5,08	0,95
5) Studies with longer follow-ups	42	4,97	1,07
6) Consideration of patient safety	41	5,21	0,96
7) Analyses of changes in health service use	40	4,65	1,24
8) Consideration of patient characteristics in telemedicine development	36	4,97	1,68
9) Methodologically rigorous RCTs	34	5,05	0,95
10) Analyses of technology acceptance	31	4,81	1,62
11) Analyses of cost-effectiveness	30	4,39	1,9
12) Abiding to reporting standards	27	4,5	1,61
13) Pragmatic study designs	26	5,11	1,83
14) Evaluation of implementation strategies	25	5,47	0,94
15) Studies with larger samples	24	4,76	1,3
16) In-depth analyses of the effectiveness of single application components	18	4,68	1,56

17) Consideration of interoperability	11	4,45	3,07
18) Inclusion of diverse study populations	10	5,30	1,14

The order of the 18 listed research needs follows the number (n) in the second column indicating how often a research need was deemed important. For each research need chosen, every participant was asked to state exactly how important s/he considers the need to be on a scale between 1 (not important at all) and 6 (very important). The mean importance is depicted in the column "mean", followed by the variance for each mean.

Table S5. Ranking of applicable pragmatic study designs.

Pragmatic designs (overall n = 26)	n
1) Waiting list control group-designs	16
2) Group-sequential designs	14
3) Select-Drop-designs	14
4) Designs employing sample-size re-estimation	13
5) Designs employing adaptive randomization	13
6) Designs employing population enrichment	8

The order of the six listed pragmatic designs follows the number (n) in the second column indicating how often a design was deemed useful for the assessment of telemedicine effectiveness. The overall n is 26 due to the fact that only those finding pragmatic designs important were presented with the list above.

Table S6. Ranking of relevant study characteristics to be reported.

Study characteristics (overall n = 27)	n
1) Data needed for effect size estimation	24
2) Information on the role of health care practitioners involved in the study	22
3) Information on how the intervention was conducted	21
4) Information on intensity of the feedback given by health care practitioners within the intervention	21
5) Information on participants' acceptance and uptake of the intervention	18
6) Data needed to adjust for baseline imbalances in clinical outcomes (e.g. HbA1c)	18
7) Reporting of theoretical basis or explanatory frameworks used in behavior change intervention	17

The order of the seven listed study characteristics follows the number (n) in the second column indicating how often a characteristic was deemed important to be reported. The overall n of 27 contains only those considering the reporting of study characteristics important.

Table S7. Results of factor analysis.

Factor	Variables	Loadings
Evaluation of effectiveness	Studies with longer follow-ups	0.77
	Methodologically rigorous RCTs	0.74
	Studies with larger samples	0.64
	Pragmatic study designs	0.57
Diversity of outcomes and standardized reporting	Analyses of cost-effectiveness	0.8
	Analyses of technology acceptance	0.69

	Abiding to reporting standards	0.44
	Inclusion of diverse study populations	0.41
	Consideration of interoperability	0.33
Research Planning	Consideration of patient safety	0.83
	Analyses of changes in health service use	0.65
	Consideration of patient characteristics in telemedicine development	0.65
Implementation science	Implementation strategies	0.87
	Evaluation of implementation strategies	0.57
User-centered design	Adequate tailoring of applications to patient needs	0.82
	In-depth analyses of the effectiveness of single application components	0.59
	Consideration of patient satisfaction	0.37
Data security	Consideration of data security	0.85

Within each factor (first column), the variables it is comprised of (second column) are listed according to the strength of their factor loading.

Table S8. Questions and items in the survey.

Topic		Questions and corresponding items
1)	Profession	*For starters, we would like to know to which professional field you belong.*<br>*If you belong to more than one professional field, please pick all of them.*<br>• Health care delivery<br>• Research<br>• Financing of health care system<br>• Approval for therapeutic measures<br><br>*Please tell us what you do in your professional field.*<br>*If, for example, you work as a doctor in the field of health care delivery, please state this.*<br>• Free text imputation
2)	Telemedicine expertise	*Do you come across the topic of telemedicine in your professional field?*<br>*Please chose all the fields in which this is the case.*<br>• List see above<br><br>*We would like to better understand in how far you have experience with telemedicine in your professional field.*<br>I have…<br>• experience in developing telemedicine interventions for the chronically ill.<br>• …experience in evaluating telemedicine interventions for he chronically ill.<br>• …experience in using telemedicine interventions in my work with chronically ill patients.<br>• …experience in counselling patients on the use of telemedicine interventions.<br>Scale: no experience at all, sporadic experience, continuous experience
3)	Telemedicine definition	*People may have different understandings of what telemedicine actually is. Such, in the following, a number of diverging definitions for telemedicine is presented.*<br>Telemedicine is…

		• ...the enhancement of hospital surgery through the use of surgical robotics.<br>• ...the utilization of information and communication technology for the delivery of health care or health education over a geographical distance.<br>• ...the use of mobile technology for the quantified self, self-optimization and health care.<br>• ...the digital storage, sharing and categorization of patient data.<br>• ...the use of artificial intelligence tools or algorithms for diagnostic and treatment decisions.
4)	Ranking and importance of future research needs	*In the following, you will be presented with some topics which could guide future research in telemedicine effectiveness and implementation.*<br>*Please select all the topics you consider important for future telemedicine research.*<br>• Implementation strategies for the telemedicine application<br>• Development of telemedicine interventions tailored to individual patient characteristics<br>• Consideration of data security<br>• Consideration of patient satisfaction with the use of the telemedicine intervention<br>• Studies with longer follow-up periods, in order to measure long-term effects<br>• Consideration of patient safety<br>• Analyses of changes in health service utilization due to the use of telemedicine<br>• Consideration of patients' characteristics in telemedicine intervention planning<br>• More rigorous RCTs<br>• Analyses of user satisfaction and technology acceptance<br>• Analyses of cost-effectiveness<br>• Adherence to reporting standards when publishing effectiveness studies<br>• Pragmatic study designs (e.g. more observational studies, RCTs with waiting list control group etc.)<br>• Evaluation of implementation strategies<br>• Studies with higher numbers of participants<br>• Improved understanding of effective telemedicine components<br>• Consideration of interoperability of technology<br>• Inclusion of more diverse study populations<br><br>*In the following, you will be presented with some topics which could guide future research in telemedicine effectiveness and implementation.*<br>*For each topic, please tell us whether you think it is of high or low importance.*<br>• List see above<br>• Ranking possible from 1 = low importance to 6 = high importance
5)	Pragmatic study designs	*You stated that more pragmatic study design should be used in future telemedicine research. In the following, a selection of pragmatic study designs is presented.*<br>*Please pick all the designs you find useful for telemedicine evaluation.*<br>• Group-sequential designs include an option to stop the trial when effectiveness or futility of the intervention is successfully

		proven, or when the safety of the participants can no longer be assured.<br>• Trials with sample site re-estimation allow to adjust the sample size during intervention time, e.g. in case of large numbers of drop-outs.<br>• Studies with a select-drop design allow to assess multiple intervention components with options to "drop losers" or "select winners" early on.<br>• Studies which employ population enrichment select only participants who can be deemed likely to benefit from the intervention.<br>• Using adaptive randomisation, researchers can shift the allocation ratio of participants to the more promising intervention components during the intervention period.<br>• In studies with a waiting list control group, participants randomized into the control group receive the intervention anyway, yet after the experimental group, in order to minimize drop-outs due to disappointment of not receiving the intervention.<br>*Explanation given on Population Enrichment: "Population Enrichments means selecting participants which already have a certain disease, run the risk of getting it or appear likely to react positively to a therapeutic measure.*
6)	Reporting standards	*You stated that a more rigorous reporting of study characteristics should be an aim of future telemedicine research. In the following, a selection of study characteristics is presented.*<br>*Please pick all the characteristics you think should be reported in telemedicine effectiveness studies.*<br>• Data needed for effect size estimation<br>• Information on how the intervention was conducted<br>• Information on participants' acceptance and uptake of the intervention<br>• Information on the role of health care practitioners involved in the study<br>• Information on intensity of the feedback given by health care practitioners within the telemedicine intervention<br>• Data needed to adjust for baseline imbalances in clinical outcomes (e.g. HbA1c)<br>• Reporting of weak or non-significant effects<br>• Reporting of theoretical basis or explanatory frameworks used in behavior change interventions
